# Whole exome/genome sequencing in cyclic vomiting syndrome reveals multiple candidate genes, suggesting a model of elevated intracellular cations and mitochondrial dysfunction

**DOI:** 10.3389/fneur.2023.1151835

**Published:** 2023-05-05

**Authors:** Omri Bar, Laurie Ebenau, Kellee Weiner, Mark Mintz, Richard G. Boles

**Affiliations:** ^1^NeurAbilities Healthcare, Voorhees, NJ, United States; ^2^NeuroNeeds, Old Lyme, CT, United States

**Keywords:** cyclic vomiting syndrome, aberrant ion gradients, mitochondrial dysfunction, cellular hyperexcitation, migraine variants

## Abstract

**Objective:**

To utilize whole exome or genome sequencing and the scientific literature for identifying candidate genes for cyclic vomiting syndrome (CVS), an idiopathic migraine variant with paroxysmal nausea and vomiting.

**Methods:**

A retrospective chart review of 80 unrelated participants, ascertained by a quaternary care CVS specialist, was conducted. Genes associated with paroxysmal symptoms were identified querying the literature for genes associated with dominant cases of intermittent vomiting or both discomfort and disability; among which the raw genetic sequence was reviewed. “Qualifying” variants were defined as coding, rare, and conserved. Additionally, “Key Qualifying” variants were Pathogenic/Likely Pathogenic, or “Clinical” based upon the presence of a corresponding diagnosis. Candidate association to CVS was based on a point system.

**Results:**

Thirty-five paroxysmal genes were identified per the literature review. Among these, 12 genes were scored as “Highly likely” (*SCN4A*, *CACNA1A*, *CACNA1S*, *RYR2*, *TRAP1*, *MEFV*) or “Likely” (*SCN9A*, *TNFRSF1A*, *POLG*, *SCN10A*, *POGZ, TRPA1*) CVS related. Nine additional genes (*OTC, ATP1A3, ATP1A2, GFAP, SLC2A1, TUBB3*, *PPM1D*, *CHAMP1*, *HMBS*) had sufficient evidence in the literature but not from our study participants. Candidate status for mitochondrial DNA was confirmed by the literature and our study data. Among the above-listed 22 CVS candidate genes, a Key Qualifying variant was identified in 31/80 (34%), and any Qualifying variant was present in 61/80 (76%) of participants. These findings were highly statistically significant (*p* < 0.0001, *p* = 0.004, respectively) compared to an alternative hypothesis/control group regarding brain neurotransmitter receptor genes. Additional, post-analyses, less-intensive review of all genes (exome) outside our paroxysmal genes identified 13 additional genes as “Possibly” CVS related.

**Conclusion:**

All 22 CVS candidate genes are associated with either cation transport or energy metabolism (14 directly, 8 indirectly). Our findings suggest a cellular model in which aberrant ion gradients lead to mitochondrial dysfunction, or vice versa, in a pathogenic vicious cycle of cellular hyperexcitability. Among the non-paroxysmal genes identified, 5 are known causes of peripheral neuropathy. Our model is consistent with multiple current hypotheses of CVS.

## Introduction

Cyclic vomiting syndrome (CVS) is defined clinically by the presence of multiple, stereotypical, and distinct episodes of nausea and vomiting, with the essential absence of these findings between episodes (1). Episodes last for a few hours to several days, and often lead to serial emergency department or hospital visits due to intense discomfort and/or dehydration. While treatable in many cases, it is not uncommon for patients to continue to suffer from ongoing vomiting episodes for many years. First described in 1861 (2), the condition is rather common as it occurs in up to 2% of school-aged children (3) and is increasingly recognized in adults (4–8). However, despite the condition’s early description and high prevalence, CVS remains under-recognized by clinicians.

Often considered as a migraine variant, CVS is defined clinically, and its etiology remains unclear. Further investigation is necessary to determine if CVS is a single entity or multiple conditions with overlapping presentations. Various papers have described many different pathways as likely being involved in its pathophysiology, including dysautonomia (9), stress response (10), energy (mitochondrial) metabolism (11), ion channels (12), neurotransmitters receptors (13), and neuropathy (4, 6). In addition, CVS shares many co-morbidities and endophenotypes with other paroxysmal disorders such as migraine (14–19), epilepsy (20, 21), and panic disorder (22). The various potential etiologies for CVS have led to a wide range of different treatments offered, including tricyclic antidepressants, beta blockers, anticonvulsants, anti-inflammatory drugs, dextrose-containing intravenous fluid, L-carnitine, and coenzyme Q10. Essentially, all common disorders have substantial genetic and environmental components to their pathophysiology. CVS clearly has an important genetic component, not in that CVS itself is often familial, although this is sometimes the case, but in that certain disease manifestations are very common among the first-degree relatives, especially chronic pain, fatigue, and other gastrointestinal and dysautonomic disorders (11, 20).

A powerful modern way to identify genetic components of any idiopathic condition is DNA sequencing, either of all ~23 K genes (whole exome sequencing, WES), or better to sequence essentially all the DNA including the vast non-protein coding regions between and within genes (whole genome sequencing, WGS). A non-directed approach that analyzes all sequence variants throughout the genome has an advantage in that disease-related variants could be detected even if current hypotheses regarding disease pathogenesis are widely incorrect. However, since tens of thousands of sequence variants per individual are identified in the exome alone, a non-directed approach requires vast resources for an in-depth sequence evaluation of all genes. A targeted approach in a much smaller subset of genes thought to be at higher risk for harboring the genetic component of disease allows for an in-depth evaluation, but is successful only if you choose the correct genes. Thus, both approaches have different pros and cons.

What quality is both sensitive and specific to CVS such that it can be used to drastically narrow down a gene list, yet still likely include many of the genes involved in its pathogenesis? Thirty years of clinical experience managing CVS patients has suggested to the Corresponding Author that the strict paroxysmal nature of CVS can define this essential quality. Episodes are generally abrupt, distinct, and stereotypical within each person, resolving completely only to reoccur again and again. While a single episode of nausea and vomiting can have any number of different etiologies, having multiple similar (stereotypical) episodes defines CVS. In this regard, CVS is not that different from some other paroxysmal neurological conditions, in particular migraine and epilepsy, for which CVS shares many features, and are often comorbid in the same individuals and families (22).

In addition to the defined characteristics of nausea and vomiting, most CVS suffers also describe a substantial degree of discomfort (always nausea, but also pain: abdominal, headache, less often myalgia) and disability (lethargy, weakness, fatigue) during episodes. Discomfort is often severe, and many patients also meet clinical diagnostic criteria for the (additionally) clinically-defined conditions of abdominal migraine and migraine headache. Disability is variable, but appears to essentially always be present, and can be severe enough to be described by patients as “conscious coma” or “paralysis.”

In this study, our hypothesis regarding the essential paroxysmal nature of discomfort and disability was used to define a gene list to query sequence data in 80 unrelated participants (75 with WES or WGS) with CVS. This was followed by a selected review of Key Qualifying variants (per *Methods*) throughout the remaining genes (exome). In addition, the literature was carefully reviewed to identify any genes published as potentially associated with paroxysmal vomiting. Comparison was made to another gene list corresponding to an alternative hypothesis involving brain neurotransmitter receptor genes, which serves as a control. The net result is the development of a comprehensive candidate gene list for CVS, from which it is hoped that a better understanding of this condition will arise, which in turn would potentially lead to improved diagnostics, therapies, and clinical outcomes.

## Methods

### Participants

Inclusion for participation in this retrospective study was based on the Rome IV diagnostic criteria for CVS,^1^ as determined by the Corresponding Author, who is a pediatric quaternary care specialist known for conducting clinical care and research into CVS. Thus, participants must have all the following manifestations: stereotypical episodes of vomiting; three or more discrete episodes in the prior year; and the absence of nausea and vomiting between episodes. For this study, participants could meet these criteria in the present or at some time in the past. For example, several participants previously had active vomiting episodes meeting Rome IV criteria (23), but have since entered clinical remission, or the vomiting episodes evolved to migraine (headache or abdominal). Only individuals who were personally evaluated in a clinical context by the Corresponding Author were included. The minimum requirements of this evaluation included a chart review, interview of the parent(s) or adult patient, and a physical examination (which in some cases was *via* teleconference). This study was approved by the Advarra IRB (human subjects committee^2^) as a retrospective chart review of clinical records already available to the Corresponding Author/treating physician. To identify all potential participants, medical records were reviewed from the previous 3 years, roughly equivalent to the time in which extensive DNA sequencing was routinely obtained by the Corresponding Author for all CVS patients. In the few cases where more than one family member met study criteria, the participant was assigned to be the proband (person first presenting as a patient). In cases of affected siblings presenting simultaneously, the elder was assigned, and in one case of twins, the more severely affected was assigned. Thus, all study participants have no known genetic relationships to each other. In this retrospective study, only patients with prior genetic sequencing were included, and all data considered for the study was available to the authors prior to 9/1/2021.

### Genetic sequencing

All genetic testing results pre-existed the commencement of the retrospective record review; no additional testing was performed for the purpose of this study. Sequencing was performed by multiple laboratories, in many cases determined by the preferences of the referring physicians for previously ordered testing, and/or the coverage by the families’ insurance company. To eliminate bias against genes previously identified as being related/potentially related to CVS, five “legacy” participants with positive results on prior panel sequencing (nucSEEK®, Courtagen, Woburn, MA) were also included in the study. Legacy patients did not receive additional genetic testing since a molecular diagnosis was obtained and thus further testing was not clinically indicated. All patients testing negative on panel testing had additional WES and/or WGS testing, except for one who was in a prolonged remission. The nucSEEK panel consisted of ~1,200 genes corresponding to known conditions and designed primarily for mitochondrial disease, but including many genes for entities often presenting similarly, including ion channelopathies. All participants also underwent sequencing of the mitochondrial DNA (mtDNA), either as an integral part of WGS, or ordered separately. Sequencing technology reveals small variants, generally on the order of less than 50 nucleotides in size. WGS automatically included detection of larger variants (e.g., deletions, duplications), and this technology was variably applied to patients receiving WES, dependent on whether large variant testing was included by the laboratory as part of WES, or upon clinical suspicion. The full sequence data on all participants was evaluated on a clinical basis, first by the laboratory and then by the Corresponding Author/treating physician as part of his routine patient care prior to the start of this retrospective study.

### Development of the paroxysmal gene list

Using the paroxysmal nature of CVS as described in the *Introduction*, our paroxysmal gene list was developed to identify genes with greater likelihood to be CVS related for more detailed analyses. The development of this list involved an exhaustive literature review across hundreds of publications using the search engines PubMed, Google Scholar, and Online Mendelian Inheritance in Man (OMIM) for any genes reported with both paroxysmal discomfort AND disability, or with paroxysmal vomiting alone, in at least three individuals (at least two of which are unrelated). Manuscripts were identified by the computerized algorithms and carefully evaluated for inclusion and exclusion criteria by an author provided with the full text. This minimalistic requirement was employed to allow for genes to be included that had not previously been highly considered for CVS involvement.

Discomfort was counted for individuals with paroxysmal nausea or pain of any kind, including headache/migraine, abdominal pain, cramps, soreness, and myalgia. In evaluating the literature for disability, it was difficult to distinguish between concepts such as lethargy, weakness, hemi/di/mono-plegia, paralysis, and stiffness, thus any of these terms were counted. The most important aspect in our review was that the clinical findings be paroxysmal. However, the presence of progressive symptomatology did not exclude a gene also associated with paroxysmal symptomatology. Synonyms for paroxysmal queried in the literature search included cyclic(al), episodic (episodes of), intermittent, occasional, periodic(al) (periods of), recurrent, recurring, repeated, frequent, serial, monthly, weekly, and sporadic. Casesassociated with fatigue without other indications of disability, or with vomiting only during seizures, were excluded. All publications that contributed to inclusion to this study are cited in the *References* and in Supplementary Table 1. Once a gene clearly met our criteria for paroxysmal discomfort and disability, literature search was often terminated, thus these references are not complete. Literature review for paroxysmal vomiting was complete with the exception of OTC in which we stopped once exceeding 50 cases (Tables 1 and 2).

**Table 1:** Our paroxysmal gene list with associated phenotypes.

Encoded protein function	Gene (with associated paroxysmal disorder phenotype per our literature review)
Ion channels/pumps	
Cations	
Sodium channels	*SCN1A* (FHM, Sz), *SCN2A* (EAx, Sz), *SCN4A* (HOKPP, HYPP), *SCN9A* (EPS), *SCN10A* (EPS), *SCN11A* (EPS)
Potassium channels	*KCNA1* (EAx), *KCNJ2* (Afib, ATS, PP), *KCNJ18* (PP)
Calcium channels	*CACNA1A* (EAx, FHM, Sz), *CACNA1S* (HOKPP, MH, PP), *RYR2* (CVS, VA)
Non-selective cation channels	*SLC1A3* (EAx), *TRPA1* (EPS)
Sodium/potassium transporters	*ATP1A2* (AHC, FBM, FHM), *ATP1A3* (AHC, Sz)
Indirect to cation transport	*CHAMP1* (CVS^a^, Sz), *GFAP* (CVS^a^), *PRRT2* (EKD, FICPC, Sz)
Anions	
Chloride channel	*CLCN1* (MC)
Mitochondrial dysfunction	
Mitochondrial enzyme related	*HMBS* (AIP), *PDHA1* (EW), *POLG* (EAx, CVS^a^)
Oxidative stress	*PNKD* (PNKD), *TRAP1* (EPS)
Impaired mitophagy	*MEFV* (FMF), *TNFRSF1A* (FPF)
Substrate availability	*SLC2A1* (EAx, FHM, Sz)
Mitochondrial mobility	*TUBB3* (CVS)
Indirectly affects respiratory chain	*OTC* (hyperammonemia), *POGZ* (CVS), *PPM1D* (CVS^a^)
Non-ion or mitochondrial^b^	*CDK8* (CVS^a^), *CNR1* (CVS^a^), *OPRM1* (CVS^a^)

Afib: atrial fibrillation, AHC: alternating hemiplegia of childhood, AIP: acute intermittent porphyria, ATS: Andersen-Tawil syndrome, EAx: episodic ataxia, EKD: episodic kinesigenic dyskinesia, EPS: episodic pain syndrome (or near synonyms), EW: episodic weakness, FBM: familial basilar migraine, FHM: familial hemiplegic migraine, FICPC: familial infantile convulsions with paroxysmal choreoathetosis, FMF: familial Mediterranean fever, FPF: familial periodic fever, HOKPP: hypokalemic periodic paralysis, HYPP: hyperkalemic periodic paralysis, MC: myotonia congenita, MH: malignant hyperthermia, PNKD: paroxysmal nonkinesgenic dyskinesia, PP: periodic paralysis (including thyrotoxic), Sz: seizures, VA: ventricular arrhythmia

a CVS refers to instances where only one publication was found to provide enough support for inclusion in the analysis, or episodes of vomiting may or may not be CVS.

b These genes were ultimately found to not be associated with CVS per Results and Discussion.

**Table 2:** Paroxysmal nuclear gene variants in our participants.

Gene	Key variants^a^	Other qualifying variants	Points by our study^b^
*ATP1A2*		(p.Glu492Lys: #1)	1
*ATP1A3*			0
*CACNA1A*		(p.Ala454Thr [x2]: #1, 11), (p.Arg68Gln: #76), (p.Arg2294Pro: #74), (p.Arg2298Pro [x2]: #43, 75), (CAG 7 [X9]: #5, 8, 27, 30, 52[HOM], 55, 67, 76, 80[HOM]), (CAG 4 [x2]: #31, 70), (p.Tyr2228Asn: #50), (p.Arg2135His: #46)	19
*CACNA1S*	(p.Ser516Leu: #30, CC: ataxia, PP), (p.Thr1335Ser: #72, CC: episodes of profound fatigue, ptosis), (p.Thr1354Ser: #16, CC: PP, hand weakness asymmetrical during migraine/CVS, frequent twitches), (p.Val1253Ala: #23, CC: PP)	(p.Ala814Thr, p.Tyr299His: #69), (p.Cys288Gly: #32), (p.Pro1839Ser: #14), (p.Ser1857Asn: #43)	17
*CDK8*			0
*CHAMP1*		(p.Pro406Ser: #25)	1
*CLCN1*	(p.Thr736Ile: #68, CC: myalgia, muscle tightness, episodes of ptosis and muscle weakness)		3
*CNR1*			0
*GFAP*		(p.Asp157Asn: #48), (p.Pro47Leu[x2]: #35, 76)	3
*HMBS*			0
*KCNA1*			0
*KCNJ2*			0
*KCNJ18*	(p.Gln407*: #37, CC: PP)		3
*MEFV*	(p.Ala744Ser: #53), (p.Ile591Thr: #65, CC: paroxysmal abdominal pain, eye pain as main prodrome of vomiting episodes), (p.Val726Ala, c.*381 T > C: #5, CC: paroxysmal severe neuropathic pain in four extremities, joint and muscle pain, migraine, abdominal pain during episodes, hives)	(p.Pro369Ser: #36, 66), (p.Arg408Gln: #66)	12
*OPRM1*		(p.Cys192Phe [x3]: #2, 34, 73), (p.Ser147Cys: #53), (p.Ser451Phe: #11)	5
*OTC*			0
*PDHA1*		(p.Met320Leu: #28)	1
*PNKD*		(p.Glu307Lys: #43)	1
*POGZ*	(p.Cys652Arg, *de novo*: #18, CC: NDD), (p.Phe806Leu, *de novo*: #77, CC: ASD, microcephaly, ID)	(p.Lys871Asn: #27)	7
*POLG*	(p.Lys460_Leu463del: #74)	(p.Arg42_Gln43insGln: #38), (p.Gly268Ala [x2]: #9, 18), (p.Gly517Val [x3]: #38, 52, 58)	9
*PPM1D*	(p.Tyr401fs, *de novo*: #56, CC: dysmorphic features, NDD)		3
*PRRT2*		(p.Pro216Leu [x3]: #41, 42, 54)	3
*RYR2*	(p.Tyr3459*: #54), (p.Glu4431Lys: #44, CC: chronic pain, GI dysmotility, fatigue; severe abdominal pain and fatigue during episodes), (p.Gly2094Ser: #53, CC: panic disorder, idiopathic urticaria)	(p.Arg3567Cys: #9), (p.Arg4573His: #35), (p.Asn4736Asp: #34), (p.Gly1885Glu: #4), (p.Ser2829Gly: #75), (p.Thr1107Met: #15)	15
*SCN1A*	(p.Arg1928Gly: #80, CC: ice pick migraine with muscle weakness)	(p.Ile1437Val, p.Ile1452Val, p.Ile1465Val: #29)^c^	4
*SCN2A*		(p.Ile403Thr, p.Thr674Ala: #58)	2
*SCN4A*	(p.Ala488Thr: #68, CC: myalgia, muscle tightness, episodes of ptosis and muscle weakness), (p.His599Arg [x2]: #61, 76, CC: PP), (p.Ser906Thr: #63, CC: PP, tremor, strabismus)	(p.Arg1408Cys: #45), (p.Lys477Arg: #49), (p.Thr323Met: #50), (p.Ser906Thr[x5]: #15, 25, 52, 73, 78)	20
*SCN9A*	(p.Asn1256Ser: #9, CC: myalgia, migraine with aura, abdominal pain, chronic daily headache), (p.Ser802Gly: #27, CC: severe migraine with aura)	(p.Val1726Phe: #7), (p.Val1715Phe: #9)	8
*SCN10A*	(p.Ala123Val: #64, CC: CRPS), (p.Arg14Leu: #47, CC: widespread pain), (p.Val1697Ile: #27, CC: severe migraine with aura)		9
*SCN11A*		(p.Gly1736Val: #50)	1
*SLC1A3*		(p.Arg47Gln: #77)	1
*SLC2A1*			0
*TNFRSF1A*	(p.Arg121Gln [x3]: #14, 60, 68)	(p.Pro75Leu: #69)	10
*TRAP1*	(p.Ile253Val: #18), (p.Arg128His: #60, CC: fatigue, pain, GI issues)	(p.Arg469Cys: #7), (p.Asp685Asn, p.Arg469His: #39), (p.Gln165Glu: #14), (p.Gly445Ser: #3), (p.Ser477_Gly478insAla [x2]: #3, 76), (p.Tyr444Asn: #51)	14
*TRPA1*	(p.Ala138Ser: #49, CC: mild NDD, chronic pain syndrome, main symptom during episodes is severe diaphragmatic pain requiring hydromorphone), (p.Met214Thr: #52, CC: episodic pain syndrome)	(p.Asn109Lys: #2)	7
*TUBB3*			0

a “Key Qualifying” variants include “P/LP” (Pathogenic or Likely Pathogenic, in red font) and “Clinical” (in purple font). “Qualifying” variants were defined as being rare and highly conserved, see Methods. Variants that were both P/LP and Clinical are colored in red.

b Per our scoring algorithm in Methods, which gave higher weight (3 points) to Key Qualifying variants versus “Other Qualifying” variants (1 point). Literature points were awarded as one per family with reported paroxysmal nausea and vomiting (CVS). Composite refers to the highest color designation of the two previous columns.

c Three closely-spaced variants with identical prevalence data and thus co-segregate.

The development of our paroxysmal gene list was designed predominantly to look for single copy (predominately heterozygous, dominantly-inherited) variants that may be risk factors for the development of CVS. X-linked recessive and mtDNA genes, including those that encode for metabolic conditions, were not excluded as they can be the equivalent of single copy. Since a single variant can result in disease, and the average person has tens of thousands of variants in their exome, a relatively small gene list was needed to allow for careful evaluation of all sequence variants therein. Autosomal-recessive metabolic conditions involve two variants each, are thus far easier to identify, and this analysis was performed genome-wide, as discussed in Data Analyses.

How to score of the 37 individual genes encoded on the mtDNA was exceptionally difficult to determine. Multiple mtDNA genes have previously been reported to be associated with CVS, yet variants in different mtDNA genes generally have highly overlapping phenotypes (24–29). Thus, it was decided to consider the mtDNA as a single gene in terms of this study.

### Development of the dominant neurotransmitter receptor *control* gene list

An excellent control for this study would be to compare variants in the paroxysmal genes between a CVS experimental group and a “normal” control group. However, the main clinical laboratories involved in this work do not have a cohort of normal controls that have underwent WES/WGS and that are available to us. Using control sequences from online/public databases would be a poor control group as we would be unable to perform identical analyses in terms of the “wet” sequencing and the “dry” variant interpretation. Thus, we chose to use our 75 CVS patients that underwent WES/WGS and perform the identical analyses in another set of genes.

We could have chosen a control gene list based on the encoded protein function being very different from those in our paroxysmal gene list, or at random. However, it would be difficult to control for bias in terms of gene size and functionality, which could affect the number and type of variants present. Instead, we chose a second potential experimental group, genes that encode for neurotransmitter receptors and result in dominantly-inherited, brain disease (see *Discussion*). Our “Dominant Neurotransmitter Receptor (DNTR) Gene List” was created using^3^ with keywords “receptor and dominant and” either “GABA,” glutamate,” or “glycine.”

### Data analyses

In addition to the official laboratory reports and clinical notes, as part of clinical care, raw genomic data from each participant was evaluated personally by the Corresponding Author. For Variantyx, this included a comprehensive review of the raw data on their bioinformatics platform accessible to laboratory personnel, including IGV of any variants of interest. For other laboratories, this included extensive review of the VCF converted to Excel format (by GeneSavvy, Seattle, WA) and linked to a variety of external databases regarding prevalence (gnomAD) (30) and evolutionary conservation (“conservation,” PhyloP). Variants lacking very high confidence regarding their external validity (unable to exclude false positives) were not included in this study. During the clinical data analysis, predicted altered protein structure (PolyPhen2, SIFT, and MutationTaster) was also queried. Any variants of potential relationship to the patient’s phenotype, including off-target or incidental findings, throughout the sequence were recorded in the patients’ individual visit encounter notes.

In the paroxysmal genes, all variants with a gnomAD allelic prevalence of less than 10% were recorded. Thus, as there are two alleles per person and given Hardy–Weinberg equilibrium, common variants present in over 19% of the general population were not recorded. To identify specific variants in paroxysmal genes that confer risk for CVS, all recorded variants that occurred in at least three study participants underwent statistical evaluation (Fisher Exact Test) using established gnomAD allele prevalence data (30).

Metabolic disorders can present with paroxysmal vomiting (31). The vast majority of metabolic disorders are inherited in an autosomal recessive manner. Autosomal recessive disease is fairly simple to identify on WES/WGS raw data analysis based upon the findings of two Qualifying variants in the same gene. A search throughout the exome (all protein-coding genes) was conducted for cases of 2+ Qualifying variants in the same gene. In all cases identified, the situation was flagged for in-depth review for potential recessive disease, with special consideration if that gene encodes a metabolic enzyme. Parental sequencing was requested as needed to determine the phase (if *in trans*, one variant from each parent, and thus consistent with recessive disease). This work was done personally by the Corresponding Author, a Metabolic Geneticist.

To determine potential additional genes potentially related to CVS beyond our paroxysmal, DNTR/control, and metabolic genes, we performed an additional analysis throughout the genome. For this analysis, we tabulated all Key Qualifying variants (see below) written in the patient encounter notes, based on review of the raw sequence data by the Corresponding Author.

Statistical analysis was performed by two-tailed Fisher Exact Test (Analyze a 2 × 2 contingency table)^4^.

### Variant categorization

We hypothesized that variants related to CVS predominantly alter the amino acid sequence of the encoded protein (coding variants) and are rare in both humans (low prevalence) and in other vertebrate species (high conservation). Coding variants include missense, nonsense, frameshift, exonic indels, and predicted splice sites (> 0.6 on SpliceRF or SpliceADA). For prevalence, we used an allelic cutoff of 2% from gnomAD (corresponding to near 4% population frequency). Regarding conservation, we deferred to the cut-off values for each laboratory corresponding to moderate or higher conservation, roughly corresponding to good conservation through mammals. For Variantyx patients, the cut-off used was PhyloP ≥1.5. The UCSC Genome Browser^5^ was used when in doubt, with the cutoff of a match in ≥90% of the mammalian species listed. All variants meeting all of the above criteria (e.g., coding, rare, and conserved) were termed as “Qualifying” variants.

Those exceptional Qualifying variants most likely to be disease related were categorized as “Key Qualifying” variants, and additionally met at least one of the following categories:

- Pathogenic or Likely Pathogenic (“P/LP”) variants: These variants are listed in ClinVar^6^ as being either pathogenic (P) or likely pathogenic (LP) for disease. In cases whereas multiple laboratories have provided conflicting interpretations of pathogenicity, the variant was labeled as P/LP only if at least half of the interpretations were P or LP. Some variants are assigned as P/LP based on classification as high prediction of loss-of-function based on protein effects (e.g., nonsense, frameshift, large deletions, etc.). Each variant of this classification earned a gene 3 points.
- “Clinical” variants: These variants were defined by the match of a clinical diagnosis related to a non-CVS paroxysmal disease. For example, a participant that meets diagnostic criteria for both CVS and episodic ataxia (EAx) has a Qualifying variant in the CACNA1A gene (a common cause of EAx). In this case, that variant is clinically determined to be disease associated (with EAx) independent of this study or the presence of CVS. In clinical genetic practice, thus Clinical variants correspond to variants of uncertain significance (VUS) thought to be very likely to be a disease related as the reported phenotype for the gene closely matches the clinical phenotype of the patient (clinical correlation). The CVS phenotype was obviously excluded to avoid a circular argument. Each variant of this classification earned a gene 3 points.

Some variants were both P/LP and Clinical, but only resulted in 3 points.

The mitochondrial DNA (mtDNA) has several differences from nuclear DNA, so some allowances needed to be made. Variants with heteroplasmy (minor allele proportion) < 20% were excluded as likely being of recent somatic origin. None of the mtDNA variants thus excluded were reported in MitoMap^7^ as associated with disease. Heteroplasmy of 40–98% was labeled as P/LP, as were some rare exceptions with very-high conservation of secondary structure as listed in the legend of Table 1. The determination of whether any mtDNA variant should be classified as a Clinical variant was difficult given the extreme protean findings associated with mtDNA. Therefore, we only counted cases that had mitochondrial-related clinical findings in four or more domains among neuromuscular, neurodevelopmental, neuropsychiatric, functional (e.g., pain, gastrointestinal, dysautonomic), endocrine, immunological, metabolic (laboratory signs of mitochondrial dysfunction), and enzymological (complex I or IV < 30% in muscle or buccal cells, the latter by MitoSwab, PA) (Table 1).

“Other Qualifying” variants included all Qualifying variants that did not qualify as Key Qualifying variants, earning 1 point each.

Once sequence data review was completed and categorized, points were tallied to determine how likely each gene is to be related to CVS:

- ≥12 points: “Highly likely” to be CVS related (red in Table 2)
- 6–11 points: “Likely” CVS related (orange in Table 2)
- 3–5 points: “Possibly” CVS related (yellow in Table 2)
- ≤2 points: “No evidence” to be CVS related (white in Table 2)

To determine the strength of the literature for CVS involvement, each gene, paroxysmal or “control”/DNTR, was scored giving each case of episodic vomiting one point per family, regardless of how many members of each family were affected.

## Results

A total of 105 unrelated patients with CVS were evaluated by the Corresponding Author within the 3-year time frame of this study. Two did not have testing as they were in prolonged clinical remission, and WES/WGS was clinically indicated in the remaining 103. Among these 103 patients, 13 decided against testing due to non-covered costs, 10 did not want did not want to pursue testing for other or unknown reasons, and 5 agreed to pursue testing but were subsequently lost to follow-up. Altogether, WES/WGS was ordered and evaluated in 75 participants (73% of those in which it was recommended).

WGS was performed on 35 participants (34 from Variantyx, Framingham, MA, USA; 1 from Centogene, Rostock, Germany). WES plus mtDNA sequencing was performed on 40 participants (26 from Centogene; 7 from GeneDx, Gaithersburg, MD, USA; and 7 from six different clinical laboratories). An additional 5 legacy participants were added whereas WES/WGS was recommended but not performed, yet previous panel testing revealed a Qualifying variant in a paroxysmal gene (see *Methods*). Altogether, these 80 patients were enrolled as our study participants.

Among our 80 participants, 46 (58%) were female, and 61 (76%) were of Western Eurasian ancestry, while another 10 participants had one parent of Western Eurasian ancestry. Thus, Western Eurasian ancestry accounted for 66/80 (83%; 61 + 0.5(10) = 66) of the genetic component of our population. Given this predominance and large degree of ancestral heterogeneity in the remainder of our patient population, we used gnomAD data for Non-Finnish Europeans (NFE) as the comparison group. Age of onset of vomiting episodes was <1 year: 14, 1–3 years: 11, 3–6 years: 7, 6–12 years: 12, 12–18 years: 21, > 18 years: 7, and not recorded: 8. Additional clinical data on our participant population will be published separately. Essentially, all participants had additional clinical symptomatology beyond CVS, which is consistent with previously reported studies regarding this condition (11). Clinical information will be described in a future publication using our current data, including with participant numbers that will correspond to the numbers in the tables of this manuscript.

As a result of our literature review, we identified 35 genes (Table 3; Supplementary Table 1) with reported paroxysmal-disease manifestations as discussed in *Methods*. One of these genes, *SCN10A*, did not meet criteria yet was included as the structurally, functionally, and clinically similar genes *SCN9A* and *SCN11A* were found to make criteria, while very little is published regarding *SCN10A*. This defines our 35-gene paroxysmal gene list for which in-depth sequence analysis was performed. As a result of our review on OMIM, we identified 20 receptor genes as discussed in *Methods*: 8 GABA (*GABBR1,2*, *GABRA1,5*, *GABRB1-3*, *GABRG2*), 4 AMPA glutamate (*GRIA1-4*), 6 NMDA glutamate (*GRIN1,A,2A-D*), 1 metabotropic glutamate (*GRM1*), and 1 glycine (*GLRA1*). This defines our DNTR gene list for which in-depth sequence analysis was performed as a control/comparison.

**Table 3:** MtDNA gene variants in our participants.

Gene	Key variants	Other qualifying variants
*ATP6*	m.8939 T > C: #53 [6 domains]	
*COX2*	m.7673A > G: #20 [6 domains], m.7761A > G: #33 [6 domains]	
*COX3*		m.9738G > A: #14 [1 domain]
*CYB*	m.15740C > T (94%): #74 [6 domains]	m.15884G > A: #12 [3 domains]
*ND1*		m.3565A > C: #42 [1 domain]
*ND5*	m.12706 T>C^1^: #39 [6 domains]	
*TA*	m.15904C>T^2^: #19 (39%) [5 domains]	
*TG*		m.10003 T > C: #51 [2 domains]
*TM*		m.4464A > G: #25 [2 domains]
*TR*	m.10410 T>A^3^: #39 [4 domains]	
*TT*	m.15907A>G^4^: #41 [4 domains]	
Multiple	del m.492–14,240 (46%): #73 [5 domains]; 7.7 kb heteroplasmic (proportion not determined) deletion from m.6468–14,148 [6 domains]	

Classification of variants (e.g., font color) as per Table 4 and Methods. Domains refer to clinical and laboratory areas meeting clinical correlation for a possible diagnosis of a mitochondrial disorder (see text), with a cutoff of ≥ 4 domains for a Clinical variant.

1 A known disease-causing variant per MitoMap, and Likely Pathogenic per ClinVar.

2 Changes a purine to a pyrimidine in a location whereas no mammalian species reported has a pyrimidine (per mitotRNAdb, http://mttrna.bioinf.uni-leipzig.de).

3 Adds a Watson-Crick bond in the ACC stem where humans generally lack this binding, yet almost all mammals (to platypus) have such binding (mitotRNAdb).

4 Loss of Watson-Crick binding in the D-stem. All mammalian species sequenced have Watson-Crick binding at this location (mitotRNAdb).

All single nucleotide variants in the paroxysmal and DNTR/control gene lists were verified with the requisite high level of confidence regarding validity, as per standard measures in the art (IGV, sequencing depth, prevalence, review of previous/relatives’ sequences, etc.). However, small (< 50 nucleotides) non-single nucleotide variants revealed less confidence, which is expected as such variants are far more likely to be false positives than single nucleotide variants. Thus, insufficient confidence was assigned to small non-single nucleotide variants that are not well-established polymorphisms (per gnomAD) in which IGV was unavailable. This resulted in the exclusion of 5 small indels (deletions and insertions) from the paroxysmal genes, all in *CACNA1A*, and one dinucleotide *GFAP* variant (c.1276_1277delACinsGT, p.Thr426Val) present in three participants. Certainly, some of these variants might be real, including 3 in *CACNA1A* that result in frameshift (thus, would be P/LP Key variants), and thus our data regarding these genes may be more significant than our paper reflects.

Among our 35 paroxysmal genes, based upon our scoring algorithm, the following 6 genes were determined to be “Highly likely” to be related to CVS: *SCN4A*, *CACNA1A*, *CACNA1S*, *RYR2*, *TRAP1*, and *MEFV* (in order of scores; marked in red, Table 2, last column). An additional 6 genes were determined to be “Likely” related to CVS: *TNFRSF1A*, *POLG*, *SCN10A*, *SCN9A*, *POGZ*, and *TRPA1* (orange, Table 2). Among these 12 genes, 31 Key Qualifying variants were identified, including 10 P/LP and 21 Clinical variants, with at least one Key Qualifying variant in 27 participants (34%, Table 2). Additionally, 88 Other Qualifying variants were identified among the paroxysmal genes. Altogether, a Qualifying variant (Key or Other) was identified in 61 participants (76%) among these 35 genes.

In the mtDNA, 10 Key Qualifying variants were identified, all of which met our criteria for Clinical variants; 6 of which were classified as P/LP (Table 1). At least one Key Qualifying mtDNA variant was identified in 10 participants (13%). Additionally, 5 Other Qualifying variants were identified in the mtDNA (Table 1). Considered as a single gene in this study, the mtDNA was determined to be “Highly likely” to be related to CVS.

A total of 138 variants among the paroxysmal genes were identified in at least three study participants, including 103 non-coding variants, 11 uncommon coding variants (gnomAD prevalence 2%–10%), 20 rare, lesser-conserved (PhyloP <1.5, or as per *Methods*), coding variants, and 4 rare, highly-conserved, coding variants (*OPRM1* p.Cys192Phe, *POLG* p.Gly517Val, *PRRT2* p.Pro216Leu, and *SCN4A* p.Ser906Thr). None of these variants was statistically related to CVS versus gnomAD data as the control.

Our literature search identified that paroxysmal vomiting (hence, likely CVS) had been reported previously regarding several genes (Table 4; Supplementary Table 1), of which 5 genes were reported in at least 12 cases, thus labeled as Highly likely to be related to CVS, and an additional 6 genes were reported in at least six cases, labeled as Likely related. As can be seen in Table 4, some of these genes overlap those that were identified in the study. However, 4 (*OTC*, *ATP1A3*, *ATP1A2*, *GFAP*) and 5 (*SLC2A1*, *TUBB3*, *PPM1D*, *CHAMP1*, *HMBS*) genes were labeled as Highly likely, and Likely related, respectively, from the literature despite a lack of sufficient evidence from our study.

**Table 4:** Genes at least likely to be related to CVS per our study and the literature.

Gene	Points by our study	Points by literature^a^	Composite^b^	Protein function
*ATP1A2*	1	21		Maintains the electrochemical gradients of Na and K ions across the plasma membrane; requires ATP
*ATP1A3*	0	26		Maintains the electrochemical gradients of Na and K ions across the plasma membrane; requires ATP
*CACNA1A*	19	14		Transmembrane pore-forming subunit of a voltage-gated calcium channel
*CACNA1S*	17	0		Transmembrane pore-forming subunit of a voltage-gated calcium channel
*CHAMP1*	1	6		Enables chromosome segregation. Associated with expression of genes involving cations and neurotransmitter transport
*GFAP*	3	18		Intermediate filaments in glia. Related to regulation of voltage-gated Na+, K+ and Ca2+ channels
*HMBS*	0	6		Intermediate step in heme production, which is necessary for cytochrome production in the mitochondrial respiratory chain
*MEFV*	12	2		Modulates innate immunity. Promotes inflammasome formation and maturation of IL-beta, which can induce TNF signaling
*OTC*	0	54		Mitochondrial matrix enzyme involved in the urea cycle to detoxify ammonia into urea for excretion
*POGZ*	7	10		Regulates mitotic progression. Interacts with CHAMP1 and thus may regulate cation transport
*POLG*	9	2		Replication and proof-reading of mitochondrial DNA
*PPM1D*	3	8		Regulates the DNA damage response through p53 & ATM. ATM affects mitochondrial homeostasis & modulates mitophagy.
*RYR2*	15	4		Stress-activated calcium channel on the endoplasmic reticulum
*SCN4A*	20	0		Transmembrane pore-forming subunit of a voltage-gated sodium channel
*SCN9A*	8	0		Transmembrane pore-forming subunit of a voltage-gated sodium channel
*SCN10A*	9	0		Transmembrane pore-forming subunit of a voltage-gated sodium channel
*SLC2A1*	0	11		Major glucose transporter across the blood–brain barrier
*TNFRSF1A*	10	0		Binds TNF-alpha, important in inflammation; involved in mitophagy and glial/neuronal excitation; binds TRAP1
*TRAP1*	14	2		Mitochondrial chaperone protein induced in times of oxidative stress; involved in mitophagy
*TRPA1*	7	0		Non-specific cation channel involved in perception of pain, cold, itch, sound, and oxygen concentration
*TUBB3*	0	9		A beta tubulin protein that forms microtubules in neurons
mtDNA	-	-		Encodes subunits of the respiratory chain

a Literature points were awarded as one per family with reported paroxysmal vomiting (CVS).

b Composite refers to the highest color designation of the two previous columns.

Altogether, adding the 12 paroxysmal genes from our study, the 9 additional genes from our literature review (associated with CVS by others), and the mtDNA, provides a list of 22 genes that are at least Likely associated with CVS (Table 4, Composite column, red or orange font).

None of the 20 DNTR/control genes met our criteria for reported paroxysmal-disease manifestations. Among these 20 genes, based upon our scoring algorithm, no genes were determined to be “Highly likely” to be related to CVS, and 1 gene was determined to be “Likely” related to CVS: *GRM1* (Table 2, *p* = 0.04 paroxysmal v. DNTR/control genes). Among these 20 genes, 2 Key Qualifying variants were identified including 0 P/LP and 2 Clinical variants, both in *GRM1*, with at least one Key Qualifying variant in 2 participants (3%, *p* < 0.0001 nuclear paroxysmal v. DNTR genes, corrected for variable numbers of genes 35 v. 20). Additionally, 19 Other Qualifying variants were identified among the DNTR/control genes. Altogether, a Qualifying variant (Key or Other) was identified in 13 participants among these 20 genes (17%, *p* = 0.004 nuclear paroxysmal v. DNTR genes, corrected).

Upon searching the exome for genes with at least two Qualifying variants, we only identified one metabolic gene, *GLS2* (Table 5, yellow, note b).

**Table 5:** Non-paroxysmal gene list key qualifying variants in our participants.

	Ion transport: *KCNK18* {3 points} (p.Tyr121fs: #27; migraine with aura)
	Energy metabolism: *GLS2*^b^ {3 points} (*in trans* p.Arg149Gln & p.Arg107Trp: #40, mitochondrial dysfunction on biochemical testing); *COQ2*^c^ (p.Arg22*: #1);
On target: Likely to be related to patient’s CVS	Peripheral neuropathy: *INF2* {5 points^a^, including from row below} (p.Leu480Gln: #4, CC: myalgia, pruritus, GERD, constipation, urinary incontinence); Multiple genes including *PMP22* {3 points} (chr17p12p12×1 [14,186,500-15,584,000], 1.4 Mb, inheritance unknown: #75); *SH3TC2* {3 points} (homozygous deletion^d^: #9, CC: CMT neuropathy and IBS); *GLA* {3 points} (p.Ala143Thr: #8, CC: peripheral/enteric neuropathy with an emphasis on pain); *KIF1B* {7 points} (p.Val1600Met: #22, CC: longstanding ankle braces for pain; p.Thr827Ile: #38; p.Ser1327Arg & p.Gly545Arg: #43; p.Val1600Met: #79)
On target: Unclear relationship to patient’s CVS	*COQ2*^b^ (p.Met128Lys: #10); *INF2* (p.Leu480Gln: #4; p.Arg478His: #27);
On or off target	*MEF2D*^e^ (p.Arg177Trp, *de novo*: #56); *ADAMTS2* (homozygous 41-bp deletion: #9)
Off target: Patient has condition, previously known to have variant	*CFTR* (p.Phe508del, homozygous: #49, CC: cystic fibrosis); *PORCN* (p.Arg232*: #6, CC: focal dermal hypoplasia)
Off target: Patient has condition, previously not known to have variant	*TNFRS13B* (p.Ala181Glu: #35, CC: common variable immunodeficiency); *CSNK2B* (p.Arg47*: #39, CC: neurodevelopmental disease), *ASMT* (chrXp22.33p22.33×1 [1,529,000-1,644,000], 115-kb pseudo autosomal region deletion: #31); Multiple genes including *IgG2* (chr14q32.33q32.33×1 [105,613,590-105,729,866], 115 kb that crosses 10 genes: #20, CC: frequent infections, resolved on IVIg); Multiple genes (15q11.2×1 [22,815,306-23,217,514], 42 kb: #6, CC: intellectual disability, but also can be explained by other variants)
Off target: P/LP variants conferring risk for conditions not seen in patient or family	*CTNNA3* (chr10q21.3q21.3×1 [66,195,323-66,324,900], 130 kb: #41; risk for cardiac arrhythmia); Multiple genes (15q11.2q11.2×1, 520 kb, maternally inherited: #26, risk for neurodevelopmental disease)

Definition of variants (e.g., font color) as per Table 4 and Methods. CC: clinical correlation.

a Points are added including additional variants shown in subsequent rows.

b This was the only gene identified in our analysis for metabolic disorders. The variants are considered as Key by virtue of having two in trans Qualifying variants in the same gene.

c The literature reports this gene as autosomal recessive, and thus the variants in this gene likely represent carrier status at most. However, COQ4 has a dominant form, thus the inclusion of these variants in this table.

d This homozygous 144-base-pair deletion in the SH3TC2 gene removes almost the entire intron, right up to the intron-exon border on the 3′ end. This should affect splicing, and thus is a good candidate for disease association via loss-of-function.

e A second de novo variant of uncertain significance was identified in the same patient with PPM1D-related disease: MEF2D c.529C > T, p.Arg177Trp. This gene encodes a transcription factor affecting dendritic spine density in a rat model (32), thus this may be incidental to her CVS.

Beyond the paroxysmal, DNTR/control, and metabolic genes, from raw data sequence analyses throughout the exome, we identified 20 Key Qualifying variants in 13 genes plus 4 contiguous gene deletions (Table 5). Among these, 12 variants in 6 genes plus 3 contiguous deletions involving multiple genes were clinically assessed to be incidental (“off target”), and probably not related to the patients’ CVS, although most of these did correspond to conditions known or identified in the participants (Table 5).

An additional 6 Key Qualifying variants in 5 genes plus one contiguous gene deletion are of potential relationship to CVS. One Key Qualifying variant in *KCNK18*, encoding a potassium transporter was elevated to “Possibly” CVS-related status (potentially “on target”) given the importance of cation transport among our paroxysmal genes (per Table 5 and *Discussion*). Additionally, four genes, plus one continuous gene deletion, are associated with peripheral neuropathy, a mechanism of potential importance to CVS (4, 6), and thus also were also elevated to Possibly related status (Table 5, genes in yellow font).

Altogether, 13 genes were determined as Possibly related to CVS: *CLCN1*, *KCNJ18*, *OPRM1*, *PRRT2*, *SCN1A* (paroxysmal genes with 3–5 points, yellow, Table 2), *GRM1* (DNTR/control gene with 11 points, per *Discussion*), *KCNK18* (exome-wide search, potentially on-target, Table 5), *GLS2* (exome-wide search, metabolic enzyme, 3 points, yellow, Table 5), *GLA*, *KIF1B*, *INF2*, *PMP22*, and *SH3TC2* (exome-wide search, neuropathy associated, 3–5 points, yellow, Table 5).

Finally, 2 Key Qualifying variants in 2 genes were of uncertain relationship to CVS. The first was a *de-novo* missense variant in *MEF2D*, a gene that encodes a myocyte enhancement factor involved in gene transcription (Table 5, note e). The second variant is a homozygous deletion in *ADAMTS2*, a gene that encodes a procollagen proteinase associated with recessive Ehlers-Danlos syndrome (EDS), dermatosparaxis type. However, the potential connections to CVS for either gene were insufficient to be scored as Possibly related.

## Discussion

### Candidate genes highly likely/likely related to CVS

Through the focus on the paroxysmal nature of CVS, 35 genes were identified in the literature, and sequence variants within were evaluated (Table 3). Our methodology strongly emphasized Key Qualifying variants among the candidate genes, as these variants are both prone to alter protein function and/or have a strong clinical association with paroxysmal disease. Among the 35 genes, 12 had sufficient evidence in our CVS participants to be considered “Highly likely/Likely” related to CVS. Moreover, our literature search revealed paroxysmal vomiting in 9 additional genes despite a lack of evidence from our study. Finally, adding the mtDNA as a single gene produces our list of 22 genes that we propose that are at least Likely related to CVS (Table 4). These genes were identified by our systematic analysis based on our main hypothesis of paroxysmal discomfort and disability in CVS, and not influenced by our additional analysis. However, our gene list is potentially biased in terms of more severe and complicated cases by virtue of ascertainment *via* a pediatric quaternary care specialist.

Those genes identified by the literature and those genes identified by our study overlap by less than half, which is not unexpected considering the striking differences in ascertainment. The literature cases are almost all from case reports, which are highly biased towards P/LP variants in rare genes, likely monogenic (disease causal), in cases that are somehow remarkable (e.g., severe CVS phenotype, syndromic), and ascertained from the wider population. Thus, the literature is biased towards rare causes, which simply may not have been identified in our participant group. On the other hand, most disease in general is polygenic and multifactorial, and most CVS is less-than-severe and non-syndromic (11). Our study concentrated on dominant variants, identifying Qualifying variants that are often non-P/LP, and likely are disease related in a polygenic/multifactorial manner. This is particularly the case with ion channel genes, in which most disease-predisposing variants in the literature are missense, non-P/LP, and likely disease predisposing versus disease causal (33). Thus, both methods of ascertainment are important when considering which genes are potentially related to CVS, and the highest determination among the two ascertainments is presented in the final column of Table 4.

Fourteen of these 22 genes can be placed into two categories based on the direct known function of the genes: cation transport (*ATP1A2*, *ATP1A3*, *CACNA1A*, *CACNA1S*, *RYR2*, *SCN4A*, *SCN9A*, *SCN10A*, *TRPA1*) and energy metabolism (*HMBS*, mtDNA, *POLG*, *SLC2A1*, *TRAP1*). *ATP1A2* and *ATP1A3* encode for energy (ATP)-requiring cation pumps. The remaining cation transport genes encode for facilitative (non-ATP-requiring) cation channels that transport calcium (*CACNA1A*, *CACNA1S*, *RYR2*), sodium (*SCN4A*, *SCN9A*, *SCN10A*), or cations non-selectively (*TRPA1*). *POLG* encodes the protein that both replicates and proofreads mtDNA. *TRAP1* is the main mitochondrial chaperone, which protects a variety of mitochondrial proteins in situations with high oxidative stress.

The mtDNA is not actually a “gene,” but a genome in and of itself that encodes 37 genes; although at a total size of 16.6 kilobases (kb), it is actually smaller than many single nuclear genes. Herein, the mtDNA is considered as a single gene as discussed in *Methods*. These 37 genes are involved in the transcription and translation of 13 polypeptides that are components of the mitochondrial respiratory chain complexes I, III, IV, and V. Multiple publications have shown associations between mtDNA and CVS (24–28).

The remaining two of the 14 genes have direct relationships that intuitively may not be obvious from a brief description of each gene. *HMBS* encodes an enzyme in the biosynthetic pathway of heme, a precursor for the cytochromes that compose the active site of mitochondrial complexes II, III and IV. *HMBS* variants can result in acute intermittent porphyria, which is associated with deficiencies in mitochondrial complexes and significantly decreased ATP production in brain and muscle (34, 35). This dominant disorder is characterized by paroxysmal neurologic changes and gastrointestinal dysfunction with abdominal pain. *SLC2A1* encodes for the blood–brain barrier glucose transporter, a protein physically located outside of the mitochondria. We consider this to be directly involved in energy metabolism as glucose is the primary substrate for the production of ATP.

The remaining nine genes have indirect relationships to the key mechanisms of cation transport or energy metabolism. Two of the genes, *MEFV* and *TNFRSF1A*, are involved in promoting inflammation. *MEFV* encodes pyrin, an innate immune sensor that triggers the formation of inflammasomes that mobilize production of inflammatory mediators (36). Aberrant activation of inflammasomes leads to excessive inflammatory and pathological autoimmune responses as seen in Familial Mediterranean Fever, a disorder that can be inherited in either autosomal recessive or autosomal dominant (AD) forms. In both forms, the full phenotype of Familial Mediterranean Fever consists of paroxysmal fever and other signs of inflammation including pain and rash (37). However, variable expressivity and non-penetrance are common, with many relatives carrying the gene mutation having only mild or absent symptomatology. Pyrin promotes the maturation of many inflammatory cytokines, including IL-1β that can induce tumor necrosis factor-alpha (TNF-α) to bind TNF receptor type-1, encoded by *TNFRSF1A* (38, 39), which is expressed by most cell types. *TNFRSF1A* variants can result in TNF receptor-associated periodic syndrome (TRAPS), an autoinflammatory disease characterized by paroxysmal fever, pain (chest, abdomen, muscles), rash and outwards signs of inflammation (e.g., conjunctivitis, periorbital edema) (40, 41). Sequence variants in both *MEFV* and *TNFRSF1A* have been associated with impaired mitophagy, which is the selective degradation of mitochondria by autophagy. In disease-causing *TNFRSF1A* variants, the autophagy system is defective leading to apoptosis and elevated production of mitochondrial-derived reactive oxygen species (ROS) (42).

Four of the genes (*CHAMP1, GFAP, POGZ TUBB3*) are reported in the literature as associated predominately with syndromes, meaning a set of specific disease manifestations especially neurodevelopmental disease with or without congenital malformations and dysmorphic features. Three of these genes, *GFAP*, *CHAMP1*, and *POGZ*, are all indirectly associated with cation transport. *GFAP* encodes a glial intermediate filament protein that is generally associated with Alexander disease, a rare AD leukodystrophy with progressive neurological disease. However, vomiting can be paroxysmal and precede other signs of neurological deterioration (43). Transgenic mice engineered to constitutively over-express wild-type *GFAP* lead to downregulation of various Na+, K+ and Ca2+ channels (including *RYR2*) (44). In an interesting connection among an inflammatory gene of the above paragraph, cations, *GFAP*, and visceral pain, anomalous TNF signaling in enteric glia cells can lead to visceral hypersensitivity, increased *GFAP* expression, and increased calcium (a cation) signaling (45–47). *CHAMP1* is associated with a dominant neurodevelopmental disorder with dysmorphic features (48). CVS is also described in 6 of 11 individuals with a pathogenic protein-truncating variant in CHAMP1 (Levy et al., 2022). Knock-out of *CHAMP1* in embryonic mouse brain caused downregulation of multiple ion channel genes, including cations (49). *POGZ* variants cause White-Sutton syndrome, yet another dominant neurodevelopmental disorder with dysmorphic features. Gastrointestinal manifestations are common, including cyclic vomiting (50–53). *POGZ* has a direct association with *CHAMP1* per GeneCards^8^, and both are involved in chromosomal segregation in mitosis (53). This association suggests that *POGZ* may have a potential indirect effect on cation transport *via CHAMP1*.

The last three genes (*OTC*, *TUBB3*, *PPM1D*) are indirectly associated with energy metabolism. *OTC*, on the X-chromosome, encodes the mitochondrial-matrix enzyme ornithine transcarbamylase in the urea cycle. The mouse model for *OTC* deficiency has decreased mitochondrial complex IV activity, decreased mitochondrial detoxification capacity, and reduced ATP levels in brain (54). Two specific variants in *TUBB3*, E410K (55) and R262H (56) have been reported in 5 and 3 cases with paroxysmal vomiting, respectively. *TUBB3* encodes for beta-tubulin, a microtubule component that enables anterograde mitochondrial transport along axons. *TUBB3* is associated with a dominant neurodevelopmental disorder with disrupted neuronal migration and disturbed axonal guidance resulting in malformations of cortical development (57). Mutations in *TUBB3* may compromise proper mitochondrial transport along neural axons (58). Cyclic vomiting was present in 8 of 13 patients carrying *de-novo* truncating variants in the *PPM1D* gene (59). These variants were all in the non-catalytic C terminus (amino acids 380–605) of the protein encoded by *PPM1D* (Wip1), and despite being truncation variants likely result in a protein gain-of-function (60). Wip1 inactivates the ataxia telangiectasia mutated (ATM) protein, and ATM has a role in promoting biosynthetic pathways that help maintain mitochondrial homeostasis (61). Thus, a gain-of-function in Wip1 is predicted to lead to mitochondrial dysfunction (62, 63).

### A cellular model for CVS: cellular overexcitation due to aberrant cation transport and energy metabolism

Thus, we have shown that all 22 of the genes determined to be at least Likely CVS related by our systematic analyses are associated with either aberrant cation transport or energy metabolism (14 directly, 8 indirectly). Many studies in the past have supported the relevance of these categories in relation to paroxysmal disorders beyond CVS. Variants in a variety of ion channels have been determined to be causal in many individuals with multiple different paroxysmal disorders, including hemiplegic migraine, epilepsy, hypokalemic periodic paralysis, myotonia congenita, episodic ataxia, long QT syndrome, and malignant hyperthermia 33, 64–71. Mitochondrial disorders related to polymorphisms of nuclear and/or mtDNA have also been associated with multiple episodic disorders such as migraine, epilepsy, and stroke-like episodes (72–75). In fact, multiple studies previously postulated CVS as likely associated with channelopathies and/or mitochondrial disease/dysfunction (11, 12, 24–27, 76–81).

The link of CVS to cation and mitochondrial function might lead one to propose two different pathways leading to the disorder, if not for the fact that these two concepts are intricately interrelated. At rest, and relative to weight, brain energy requirements are far and above compared to that of all other organs systems. Most of the energy produced in the brain is dedicated to fueling neuron and astrocyte ion gradients (82). Once energy metabolism fails, ion gradients fall rapidly, leading to cellular dysfunction and death. This is indeed the cause of morbidity and mortality with any anoxic condition (which prevents most energy metabolism) involving the brain, including stroke and drowning. On the other hand, gain-of-function variants in ion channel genes, the most common mechanism leading to paroxysmal disease [essentially a “leaky” channel; (83–85)], put an undue strain on energy metabolism in the requirement of ATP to constantly pump ions against the leak to maintain homeostasis. Indeed, any serious disruption to either ion channels or energy metabolism is highly predicted to affect the other.

**Figure 1:**
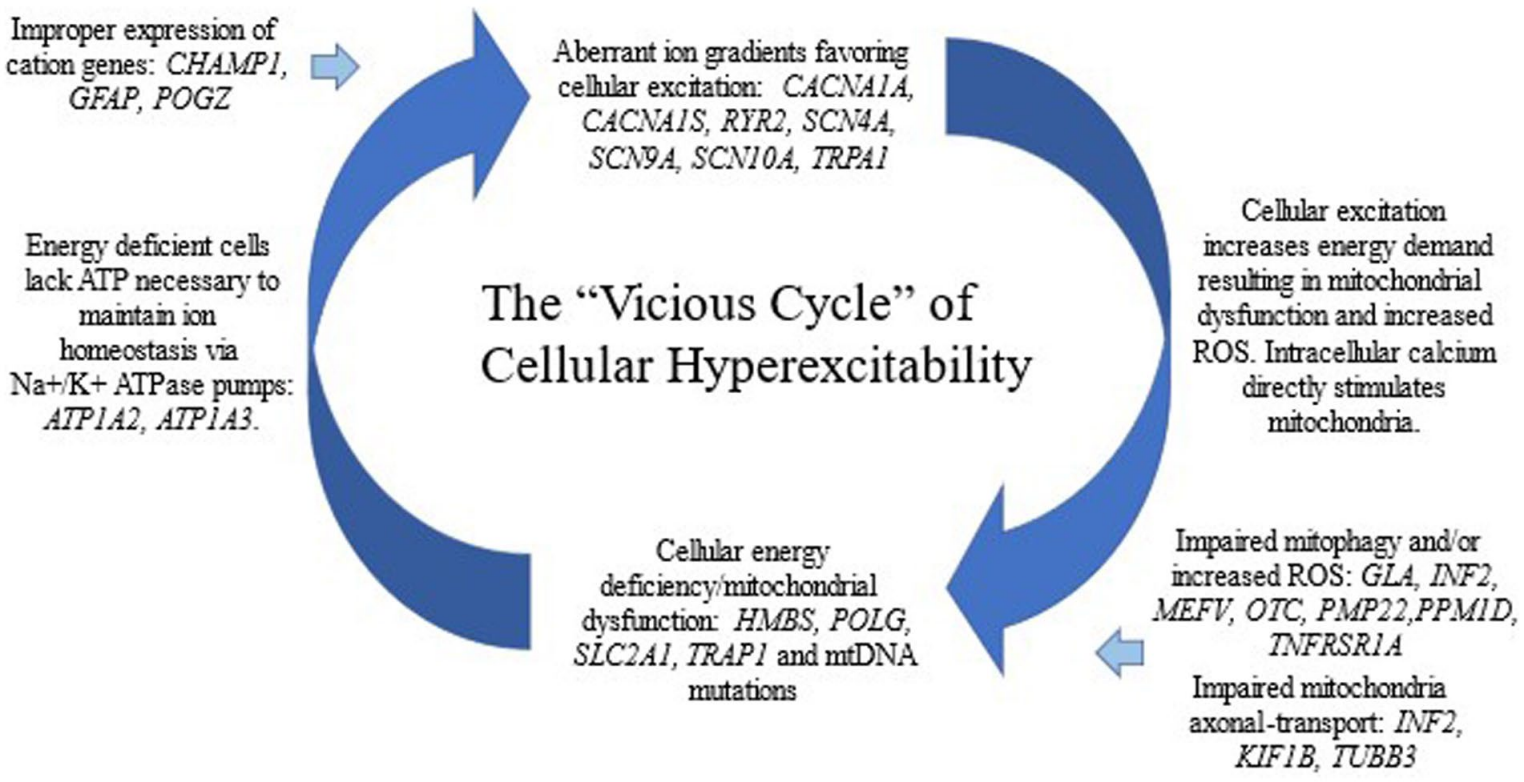
Our cellular model for CVS.

We propose a cellular model of CVS in which an aberrant ion gradient begets mitochondrial dysfunction which begets further anomalous ion gradients, etc., forming a pathogenic vicious cycle leading to cellular hyperexcitability (Figure 1). The concept of neuronal hyperexcitability has been previously proposed for CVS and has served as a possible link with several other episodic CNS disorders (86–88). In general, cellular excitation is marked by cation fluxes across the plasma membrane. The ion transport variants in our study are almost all missense variants leading to single amino acid substitutions in cation channel genes. Thus, they are likely gain-of-function variants in which a background cation leak leads to cellular hyperexcitability, as generally is reported in other dominantly-inherited channelopathies (85). These “primary” channelopathies (genetic component being in an ion channel gene) place a strain on energy metabolism, likely resulting in mitochondrial dysfunction, not only from the requirement to pump ions against the leak, but also from secondary effects on increased energy metabolism due to multiple aspects of cellular hyperexcitability. On the other hand, a “primary” defect in energy metabolism (mitochondrial disease, genetic component being in a gene involved in energy metabolism) will result in delayed homeostasis of the ion gradient in active neurons and their associated glia due to under function of the ATP-dependent cation pumps. Indeed, two of the genes encoding the main ATP-dependent cation pumps, *ATP1A2* and *ATP1A3*, are in our paroxysmal gene list and were determined to be Highly likely to be CVS related due to multiple case reports in the literature associated with intermittent vomiting.

Regarding cations, why do anomalies in sodium and calcium channels cause such similar disorders? One possible answer is that increased intracellular concentrations of either cation can lead to cellular hyperexcitability. Another possibility is that there are multiple membrane ion exchangers, including the Na + Ca2+ exchanger which can increase calcium when sodium is elevated, such that a substantial increase in any one cation is likely to affect other cations.

### Our DNTR/control genes and potential relationship to CVS

While our primary hypothesis underlying this study is that CVS is related to genes associated with other paroxysmal conditions, another potential hypothesis is that CVS is associated with genes encoding brain neurotransmitter receptors, particularly of GABA and/or glutamate. GABA receptors are anion (chloride) channels, while NMDA and AMPA ionotropic glutamate receptors are cation (calcium, sodium, and potassium) channels; NMDA receptors are also glycine receptors. There are 18 genes that encode these receptors and are associated with dominant disease per OMIM, and these became the bulk of our DNTR/control gene group. These receptors are in large part responsible for overall brain excitatory and inhibitory neurotransmission, and thus constitute a reasonable hypothesis for CVS association. In addition, being ion channels, these genes constitute an excellent group for which to compare variant analysis with our paroxysmal gene list. To bring the control list to 20, we added *GRM1*, encoding a metabotropic glutamate receptor, and *GLRA1*, encoding a glycine receptor and chloride channel. However, with the exception of two Clinical variants associated with *GRM1*, we failed to identify any Key Qualifying variant in the 20 genes. This does not rule out a role of these genes in CVS, but it does make it unlikely that primary variants in these genes are commonly associated with that disease.

*GRM1* encodes a metabotropic glutamate receptor, which is a very different protein than the ion channels common in our paroxysmal genes and composing all of our other DNTR/control genes. The GRM1 protein is not an ion channel but a G protein-coupled receptor. While the primary reported phenotype associated with the GABA and ionotropic glutamate receptor genes are epileptic and/or developmental encephalopathy, *GRM1*-associated disease results in spinocerebellar ataxia and other aberrations of movement. Qualifying variants in *GRM1* were identified in 7 of our participants. Among these, participant #17 had episodes of dizziness in the morning resembling ethanol intoxication in terms of thinking, speech, and walking, and participant #30 had ataxia during vomiting episodes. Both were assigned as Key Clinical variants, although clinical correlation is somewhat borderline. Having originated from our DNTR/control gene group, the authors felt that it was not appropriate to raise the *GRM1* gene into the Likely CVS-related category, but instead was elevated to Possible CVS association.

### Metabolic disorders and potential relationship to CVS

While metabolic disorders can present with paroxysmal vomiting (31), only a single potential case was identified by our methodology, compound heterozygous (*in trans*) Qualifying Variants in *GLS2*, which encodes for glutaminase 2. This predominantly-mitochondrial matrix-located enzyme regulates cellular energy metabolism by increasing production of glutamate and alpha-ketoglutarate (a Krebs cycle intermediate), and results in enhanced mitochondrial respiration and ATP generation. Sequence variation in this gene has not previously been linked to disease. This patient was treated with oral alpha-ketoglutarate, which anecdotally was associated with significant improvement in mood, fatigue, and pain in a dose-dependent manner. While this case is of interest as a potential novel and (anecdotally) treatable disorder, our data suggest that autosomal recessive metabolic disorders are not a common cause of CVS.

The *OTC* gene is on the X chromosome. While no evidence of CVS association was obtained from our participants, *OTC* did have the highest numbers of points of any gene (Table 1), completely derived from our literature review. *OTC* deficiency certainly qualifies as a “severe” metabolic disorder, as episodes of nausea and vomiting are generally accompanied by profound neurological dysfunction, including ataxia, altered mental status, and/or psychosis, which untreated can be fatal. This phenotype is classically reported in female heterozygotes, but also occurs in males with variants allowing some residual enzymatic activity. In general, the presence of substantial neurological/psychiatric dysfunction during vomiting episodes can be an important marker to the potential presence of an underlying metabolic disorder, including OTC deficiency, in cases with cyclic vomiting.

### Genes not in the above categories and potential relationship to CVS

The main limitation of using the paroxysmal, or any, model of CVS to determine a gene list for in-depth analyses is that there may be genes that do not correspond to the hypothesis, but nonetheless constitute some of the genetic predisposition towards the development of disease. To identify such potential genes for additional hypothesis building, Key Qualifying variants in the remaining ~23 K gene exome were queried. As these variants were all recorded in the clinical notes of each patient by the Corresponding Author, obviously this post-analysis was biased in terms of the plausibility of disease relationship based upon preconceived concepts of CVS pathophysiology in the literature and by that clinician. However, an honest attempt was made to record all sequence variants that might be disease related based upon the characteristic of the variant (e.g., P/LP) or upon clinical correlation with any condition in the patient.

Beyond ions and energy, one of the preconceived biases (e.g., hypotheses) of the Corresponding Author is that CVS may be, in part, a peripheral neuropathy. This is not a new hypothesis and is supported by the generally efficacious use of the drug amitriptyline in the treatment of CVS, as will be elaborated on later. Furthermore, peripheral neuropathy commonly results in pain, of which abdominal pain attributed to the viscera is a common feature in CVS, and pain elsewhere (headache, myalgia) is common (11). Peripheral neuropathies are generally not highly intermittent in nature, and thus their associated genes, as expected, did not appear in our paroxysmal gene list. Five different non-paroxysmal genes associated with peripheral neuropathy had at least one Key Qualifying variant in our participants (Table 5). These 5 genes were labeled as “Possibly” related to CVS (yellow, Table 5). Three of the 5 genes (*INF2*, *KIF1B*, *PMP22*) are associated with AD Charcot–Marie-Tooth (CMT) neuropathy. *SH3TC2* is associated with autosomal recessive CMT and AD median nerve neuropathy. *GLA* is on the X-chromosome, and is typically recessive in males, although females can be affected and thus likely there are dominant effects. Variants in this gene cause the lysosomal storage disorder, Fabry disease, which is characterized by a small-fiber peripheral neuropathy, among other abnormalities. Interestingly, five of our participants with Qualifying variants in peripheral neuropathy-related genes have substantial peripheral pain, consistent with CMT (Table 5).

*INF2* has direct connections with energy metabolism in its critical role in regulating mitochondrial stress in a high oxidative stress environment (89). *INF2* acts on actin barbed ends (90), a step required for actin to enable mitochondrial mobility and dynamics (fission/fusion) (91). Defects in mitochondrial dynamics disrupt the allocation of mtDNA throughout the network and subsequently lead to mitochondrial dysfunction and increased ROS. Three of the 4 other peripheral neuropathy-related genes (*PMP22*, *KIF1B*, *GLA*) also have shown to contribute to aberrant mitochondrial dynamics, axonal transport, and/or mitophagy (92–96). In fact, previous authors have postulated that CMT neuropathy in general may have a mitochondrial etiology, *via* impaired mitochondrial mobility (97). Thus, our potential finding of peripheral neuropathy-related genes in CVS fits well into our ions and energy cellular model.

A hypothesis presented by others is that CVS may be, in part, due to sequence variants in the genes encoding receptors mostly known through their binding of exogenous drugs often used to treat acute CVS episodes, but also *in vivo* bind endogenous ligands important in neurotransmission. (13) reported statistically-significant associations between CVS and three common single nucleotide polymorphisms (SNPs) in two neurotransmitter receptors genes: rs806380 and rs806368 in a cannabinoid receptor (*CNR1*) and rs1799971 in an opiate receptor (*OPRM1*). Statistical significance for these SNPs was achieved versus a control population obtained from 1,000 Genomes (Project Phase One: 1,092 individuals, as appropriate for when the study was conducted). Our data, with far fewer subjects, failed to replicate the statistical significance with *OPRM1* [11 G vs. 53 A alleles, *p* = 0.41, odds ratio 0.93, 95% C.I. 0.69–2.53, versus gnomAD in non-Finnish Europeans (NFE)]. The *CNR1* SNPs were not discoverable in our data by our methodology. However, all three SNPs vary dramatically in allele prevalence among the major world racial/ethnic groups per gnomAD. For example, *OPRM1* rs1799971 is present in 3% of African Americans (AA), 14% of Europeans (NFE), 17% of Latinos, and 38% of East Asians. Thus, small differences in racial compositions between experimental and control groups can cause spurious statistical outcomes. Using current gnomAD data (v3.1.2 controls/biobank: 7,756 NFE, 9,102 AA individuals) to construct a population matching their CVS population (91% “White” as NFE, and 9% AA), none of the three SNPs achieve statistical significance by our reanalysis (*CNR1* rs806380, *p* = 0.33; *CNR1* rs806368, *p* = 0.16; *OPRM1* rs1799971, *p* = 0.93). These two genes were added to our paroxysmal gene list based upon Wasilewski et al. (13) and our statistical analysis of their data, which would have disqualified this status, was performed after our data analysis. In our study data, we found no Qualifying variants in *CNR1*. Thus, we assert that the data does not support a significant role for *CNR1* variants in CVS at this time. We did identify 5 Other Qualifying *OPRM1* variants in our CVS population (Table 2), and thus this gene is Possibly related to CVS by our algorithm.

Certainly, we may have missed a gene or mechanism completely outside of the current hypotheses of the pathophysiology of CVS, as in-depth variant analysis throughout the entire genome is far beyond the scope of this study. Note that our study did evaluate for large genomic rearrangements (e.g., deletion, duplications) affecting paroxysmal and non-paroxysmal genes. A few were found and listed in Tables 1, 2, and 5 among the far more numerous small, mostly single-nucleotide, variants.

## Conclusion

We propose a cellular model for CVS, a vicious cycle of increased intracellular cation and energy deficiency leading to cellular hyperexcitability, which explains a wide diversity of clinical and laboratory findings regarding this disorder. We present 22 genes (11 Highly likely and 11 Likely) to be related to CVS, based either upon the systematic aspect of our study and/or our literature review (Table 4). One potential limitation of our conclusions is that our gene list was identified using the clinical hypothesis of paroxysmal discomfort and disability. Indeed, one alternative hypothesis, that CVS is associated with dominantly-inherited brain neurotransmitter receptor genes, was evaluated in parallel and found to have a far fewer (and highly statistically significant) Key and total Qualifying variants among the gene lists. The same virtually negative results were obtained for recessively-inherited metabolic disorders. Despite the biases and limitations, generating additional hypotheses regarding the pathophysiology of CVS is the main reason for our genome-wide post-analysis. In this analysis, we identified some additional genes with less robust associations (Table 5), supporting a potential role of peripheral neuropathy in CVS, but is also consistent with our vicious cycle model. Additional studies utilizing exome/genome-wide sequence data in CVS, based on yet other hypotheses or exome-wide, may reveal additional associated genes.

Most of the early, and much of the contemporary, literature consider CVS to be a migraine variant for many reasons, including common comorbidity and efficacious response to the same medications. Our model accounts for this in that many forms of migraine are associated with aberrant cation gradients and/or energy deficiency (98). Physiological (e.g., viral illness) and psychological (e.g., strong emotions) stress are known triggers of CVS (and migraine) episodes. However, stress induces cation fluxes [especially *via* the *RYR2* and *TRPA1*; (99, 100)] and places additional demands on energy metabolism, thus exacerbating mitochondrial dysfunction. Our model also accounts for the efficacy of commonly-used treatments of CVS, including mitochondrial-targeted therapies, propranolol (beta blockade interferes with catecholamine-induced release of calcium *via* the *RYR2* calcium channel (101), and amitriptyline (used for peripheral neuropathy). Episodes of CVS are abrupt and distinct, in that there is a clear onset of each episode, and not a gradual progression. This almost invariable fact about the disease fits well with a vicious cycle, in which once the cycle starts, it continues.

Indeed, one aspect of the disease that our model does not account for is how the body ever reestablishes homeostasis to stop the cycle, but this information might be very helpful in terms of therapy to terminate existing cycles. Another observation poorly explained by our model is that in many sufferers, CVS episodes are regular by the calendar, both in terms of both time of the day regarding symptom onset (circadian cycle), and for time of the month/year regarding episode intervals (infradian cycle) (3, 71, 102–104). Interestingly, much of the biological clock is present physically in the mitochondria (105, 106). More research needs to be done to fully understand this disease. Our model of “cellular hyperexcitability” begs the question of: in which cells? The question of whether CVS is fundamentally a neurological or gastrointestinal disorder is over a century old (107). Based upon the expression pattern of the candidate genes from this paper, we hypothesize the answer may be placed in vagal afferents and/or associated cell types, as will be discussed in a follow-up paper.

Genetic sequencing is not solely a research tool for CVS but is employed on a daily basis by the Corresponding Author in the clinical management of the condition in his patients, generally involving WGS as it also includes useful information in the mtDNA sequence, genetic architecture (large rearrangements), and pharmacogenomic variants (e.g., in dosing amitriptyline). Overall, 27/80 (34%) of our participants were diagnostic, defined by finding at least one Key Qualifying variant in one of the 22 main (Table 2) genes. In these patients, it can be said that the DNA analysis provided results which are more-likely-than-not related to disease. Extending to Other Qualifying variants, 61/80 (76%) of participants presented with at least one Qualifying variant in these genes. Adding in the 13 “yellow” genes with less-robust evidence of CVS association (Tables 2, 5), and 71/80 (89%) of participants presented with at least one Qualifying variant. In these patients, the determination of whether this information is clinically useful is based on whether it relates to actionability in terms of disease management, which will be a main focus of further papers. Polygenic disease appears to be common, and at least two Qualifying variants in the larger gene list of 35 genes were identified in 41/75 (55%) participants.

In the *Introduction*, we wrote that it is unknown whether CVS is a single entity or multiple conditions with shared presentations. Based upon the data in the study, we propose that CVS is likely the result of cellular hyperexcitability, as a result of a vicious cycle of elevated intracellular cations and mitochondrial dysfunction (Figure 1). Thus, with a common pathophysiology, it could be considered a single condition, although individual patients have different genetic predispositions that feed into this vicious cycle (Figure 1).

## Data availability statement

The datasets presented in this article are not readily available because of ethical and privacy restrictions. Requests to access the datasets should be directed to the corresponding author.

## Ethics statement

The studies involving human participants were reviewed and approved by Advarra IRB. Written informed consent from the participants’ legal guardian/next of kin was not required to participate in this study in accordance with the national legislation and the institutional requirements.

## Author contributions

OB performed the literature review, data collection, statistical analyses, produced the figures, and co-wrote the Discussion. LE performed data collection, produced datasets, and had primary responsibility for the Tables. KW co-authored the IRB protocol, performed data collection, produced datasets, and contributed to the Introduction. MM obtained funding and oversaw project execution. RB conceived the project, co-authored the IRB protocol, drafted the Methods, and co-wrote the Discussion. All authors contributed to the article and approved the submitted version.

## Conflict of interest

RB is an officer and receives equity from NeuroNeeds, a company that produces dietary supplements for neurological conditions.

The remaining authors declare that the research was conducted in the absence of any commercial or financial relationships that could be construed as a potential conflict of interest.

## Publisher’s note

All claims expressed in this article are solely those of the authors and do not necessarily represent those of their affiliated organizations, or those of the publisher, the editors and the reviewers. Any product that may be evaluated in this article, or claim that may be made by its manufacturer, is not guaranteed or endorsed by the publisher.

## Footnotes

^1^ https://theromefoundation.org/rome-iv/rome-iv-criteria/

^2^ https://www.advarra.com/

^3^ https://OMIM.com

^4^ https://graphpad.com

^5^ https://genome.ucsc.edu/cgi-bin/hgGateway

^6^ https://www.ncbi.nlm.nih.gov/clinvar/

^7^ www.mitomap.org/MITOMAP

^8^ https://genecards.org
